# Biallelic TYR and TKFC variants in Egyptian patients with OCA1 and new expanded TKFC features

**DOI:** 10.1186/s12864-024-10705-4

**Published:** 2024-09-09

**Authors:** Engy A. Ashaat, Nora N. Esmaiel, Sonia A. El-Saiedi, Neveen A. Ashaat, Dalia Farouk Hussen, Abeer Ramadan, Mohamed Ahmed Al Kersh, Nirvana S. AbdelHakim, Ibrahim Said, Ammal M. Metwally, Alaaeldin Fayez

**Affiliations:** 1 Clinical Genetics Department, Human Genetics and Genome Research Institute, National Research Centre, Cairo, Egypt; 2 Molecular Genetics and Enzymology Department, Human Genetics and Genome Research Institute, National Research Centre, Cairo, Egypt; 3 Pediatric Cardiology Department, Cairo University, Cairo, Egypt; 4 Professor of Genetics, Ain Shams University, Cairo, Egypt; 5 Human Cytogenetics Department, Human Genetics and Genome Research Institute, National Research Centre, Cairo, Egypt; 6 Orthopedic Department, Ain Shams University, Cairo, Egypt; 7 Fetal Medicine Unit, Faculty of Medicine, Cairo University, Cairo, Egypt; 8 Community Medicine Research Department/Medical Research and Clinical Studies Institute, National Research Centre (Affiliation ID: 60014618), Cairo, Egypt

**Keywords:** Biallelic variants, Expanding the phenotypic *TKFC* spectrum, Oculocutaneous albinism (OCA1), *TKFC*, *TYR*

## Abstract

**Background:**

Oculocutaneous albinism type1 (OCA1) is caused by the TYR gene's homozygous and compound heterozygous variants. *TKFC* gene variants cause triokinase & FMN cyclase deficiency syndrome with variable multisystemic disorders.

**Objectives:**

To determine the potential disease-causing variants in two deceased patients presenting atypical OCA1 features by demonstrating three generations for a single family. The two deceased neonates had severe skeletal abnormalities and fatal hypertrophic cardiomyopathy. We also explored the potential mechanisms for the causative relationship between *TKFC* and multisystem disorders.

**Patients and methods:**

Due to the new emerging symptoms that weren’t reported before with the TYR gene, the following methods were performed: Sanger sequencing for the TYR gene, followed by whole exome sequencing, co-segregation, and computational analyses.

**Results:**

Extensive parental consanguinity was found, and consequently an autosomal recessive mode of inheritance was prioritized. Upon performing sequencing and segregation data, the following has been confirmed: positive co-segregation of nonsense homozygous NM_000372.5:c.346C > T p.(Arg116*) variant in *TYR* gene and multisystem disease-missense homozygous NM_015533.4:c.598G > A p.(Val200Ile) variant in *TKFC* gene in the two affected index patients who deceased due to hypertrophic cardiomyopathy. Using computational analysis, we found that c.598G > A p.(Val200Ile) pathogenicity has led to the failure of L2-K1 active site closure due to the potential differential fluctuation between valine and isoleucine residues. Subsequently, disruption of endogenous DHA phosphorylation was found. Two potential mechanisms exploring the causative relationship between *TKFC* gene and multisystem disorders have been suggested.

**Conclusions:**

This study presented a first family with the co-existence of biallelic variants in TYR and TKFC genes associating severe skeletal abnormalities and lethal hypertrophic cardiomyopathy. Neither of these genes would have been pursued in the standard genetic counseling. Such discovery is paving the way for more efficient genetic counseling. Comparing *TKFC* results with literature data showed that our relevant expanded *TKFC* variant is the 3rd worldwide.

## Introduction

Albinism can be divided into ocular albinism (OA) and oculocutaneous albinism (OCA), where both OA and OCA are heterogeneous genetic disorders of melanin synthesis. According to the affected genes, OCA has differentiated into syndromic and nonsyndromic OCA [1]. Numerous phenotypic subtypes represent nonsyndromic OCA including; type 1 (OCA1, MIM#203100) which is caused by variants in the tyrosinase (*TYR*) gene (11q14–q21), type 2 (OCA2, MIM#203200) which is caused by variants in the *OCA2* gene (15q11.2–q12), type 3 (OCA3, MIM#203290) which is associated with variants in the tyrosinase-related protein gene (*TYRP1*, 9p23) and OCA type 4 (OCA4, MIM#606574) which is caused by variants in the membrane-associated transporter gene (*MATP*, 5p13.3) [2]. OCA1 is the most commonly observed in Caucasians. It accounts for the most severe OCA subtypes representing 50% of all cases worldwide [3, 4]. Syndromic OCA is caused by variants in other genes such as *HPS1, AP3B1, HPS3, HPS4, HPS5, HPS6,* and *DTNBP1 * [1]*.*

The *TYR* gene encodes a copper-containing enzyme called tyrosinase, which is included in the melanin biosynthesis pathway in hair follicles, skin, and eye melanocytes [5]. Biallelic *TYR* variants cause OCA1 with a complete or partial lack of melanin [6].

*TKFC* encodes a bifunctional enzyme, which is annotated as a homodimeric triokinase (fructose metabolism) and FMN cyclase (cyclic flavin mononucleotide, cFMN). This enzyme catalyzes the ATP-dependent phosphorylation of the trioses D-glyceraldehyde (GA) and dihydroxyacetone (DHA), and the cyclizing lyase splitting of FAD to AMP and riboflavin cyclic-4',5'-phosphate [7]. Both triokinase and FMN cyclase are annotated by residues 1–339 in the DHA kinase (K) domain and residues 359–575 in the FMN lyase (L) domain respectively. These two domains are connected by a linker region from residues 340–358 as a functional homodimer [8].

Recently, it was found that some *TKFC* variants are associated with multisystem diseases. Wortmann et al. [8], revealed that the pathogenicity of biallelic *TKFC* variants is associated with marked clinical variability, including cataracts, liver dysfunction, microcytic anemia, developmental delay, cerebellar hypoplasia, and fatal cardiomyopathy with lactic acidosis [8].

In this study, we presented two affected neonates with consanguineous parents. They were concerned with obvious OCA traits combined with marked non-OCA clinical variability. The two cases were presented by severe skeletal abnormalities, fetal cerebral hypoplasia, fetal reduced movements, liver dysfunction, and fatal hypertrophic cardiomyopathy.

This study was done to determine the potential disease-causing variants in two deceased neonates presenting atypical OCA1 features by demonstrating three generations for a single family, and to explore the potential mechanisms for the causative relationship between *TKFC* and multisystem disorders. Using sequencing techniques, co-segregation, and computational analyses, we identified co-existence variants in two different disease-associated genes, *TYR* and *TKFC*. This study represents supportive evidence for the probable occurrence of different diseases in a single patient owing to parental consanguinity with expanding of the phenotypic spectrum of TKFC.

## Patients and methods

### Ethics approval

This study was performed in line with the principles of the Declaration of Helsinki (2013) [9]. Approval was granted by the Medical Research Ethics Committee of the National Research Centre (NRC) with the registration approval number of (350) [2-3-9; 2-3-7]. The conduct of the study complied with the International Ethical Guidelines for Biomedical Research Involving Human Subjects [10]. The information disclosure for “Making sure patients understand” was guaranteed according to the recommendations of the Egyptian patients and guardians’ perception that clinical informed consent is the preferred purpose for Informed Consent practices [11].

### Patients enrolled

A family with a history of recurrent neonatal deaths and positive consanguinity were presented to the Multiple Congenital Anomalies (MCAs) Clinic, Clinical Genetics Department, National Research Centre, Egypt. 12 individuals of the family across 3 generations have been enrolled in this study.

### Clinical examination and laboratory investigation

External clinical examination, Anthropometric measurements, Echocardiography (Echo), and X-rays have been conducted for the available neonates before death.

The family history and clinical manifestations were done to evaluate identified variants concerning their disease causality. The clinical information included the following clinical features: abnormalities of the skeletal system; Absent radius; Albinism; Arthrogryposis-like, hand anomaly; Bilateral talipes equinovarus; Cardiomegaly; Hypertrophic cardiomyopathy; Chest tightness; Distal arthrogryposis.

The following laboratory investigations were detected: Increased blood urea nitrogen; Elevated circulating C-reactive protein concentration; Elevated circulating creatinine concentration; Frog-leg posture; Hand clenching; Ocular albinism; Prolonged prothrombin time; Talipes), and positive consanguinity parents.

### Cytogenetic studies

Conventional cytogenetic analysis (CCA) of peripheral blood chromosomes was performed for the 2 sibs to detect any numerical or structural chromosomal aberrations [12]. Cytogenetic nomenclature was reported according to the International System for Human Cytogenomic Nomenclature [ISCN] [13].

### Sanger sequencing

Sequencing *TYR* and *TKFC* primers were designed with Primer3 software [14].

as Forward-TYR was 5'-CTGGCCATTTCCCTAGAGC-3',

Reverse-TYR was 5'- CCACCGCAACAAGAAGAGTC-3',

Forward-TKFC was 5'- AGAGTCTGGCAGCCTTTCTC -3',

and Reverse-TKFC was 5'- GCAGGTAGAAATCAGGCAGG-3'.

Polymerase chain reaction amplicons were purified with ExoSAP, and followed by sequencing with BigDye Terminator v3.1 (Applied Biosystems, Foster City, CA, USA). Sequence chromatograms were visualized using the DNABaser tool (www.DnaBaser.com). The variants were detected by automatic and manual inspection. Sanger sequencing was used in the initial *TYR* sequencing. The confirmation and the subsequent familial segregation analysis of the detected *TYR* and *TKFC* variants was performed.

### Whole-exome sequencing

Whole exome sequencing was conducted to interpret the observed variable patients' clinical traits. All the described variants were annotated using chromosomal descriptions and were checked by LUMC mutalyzer v. 3.0.4 according to GRCH38 human genome assembly. All mentioned genes were described according to HGVS nomenclature.

Genomic DNA was enzymatically digested. The target regions were enriched using DNA capture probes including the human coding, and the mitochondrial genome. The generated library was sequenced on an Illumina platform, and aligned to the UCSC human genome GRCh37/hg19. The GATK pipeline was used to verify the detected variants. Annotation of variants is done using the BaseSpace Variant Interpreter Server. Identified variants are checked against public genetic databases; Genome Aggregation Database, 1000 Genomes, and dbSNP. Variants with a minor allele frequency of less than 1% in the gnomAD database and disease-causing variants reported in HGMD and ClinVar databases are evaluated and sorted. The investigation for relevant variants is focused on clear gene-phenotype evidence with emphasis on the autosomal recessive inheritance pattern. The copy number variation (CNV) and the mitochondrial deletions were considered.

### Computational analysis

The prioritized *TYR* and *TKFC* variants were screened using SIFT, PolyPhen, CADD, REVEL, MetLR, MutationAssessor, MutationTaster (MT), and aggregated prediction scores according to each pathogenicity index cut-off score. The curated data obtained from these tools were further analyzed using DEOGEN2, SABLE, and MutPred to identify the relevant molecular mechanisms, relative solvent accessibility (RSA), and their pathogenicity respectively. KEGG pathways analysis was used to elucidate all *TKFC*-involved pathways. GeneMANIA and STRING tools were used to resolve interactions and shared domains between *TYR* and *TKFC*. *TKFC* was homology modeled by Phyre^2^ and AlphaFold DB tools. QMEANDisCo tool was used to ensure the quality of the modeled TKFC. The YASARA tool was used to refine and energy minimization of the wild and mutant *TKFC* models. Swiss PDB viewer and CAPSflex were used to compute and predict the fluctuating amino acids within a secondary structure of *TKFC*. HPO identifiers of the observed clinical features were identified by the Phenomizer tool to validate the observed clinical features' nomenclature.

## Results

### The clinical report

According to the parents’ history; they had two neonates (III-1 and III-2) who died shortly after birth with hypertrophic cardiomyopathy without phenotypic features of the OCA.

The two enrolled neonates in this study; the 3^rd^ (III-3, male) and 4^th^ (III-4, Female) neonates were born for two consecutive years and were examined before death. Fetal ultrasonography (U/S) of the affected male was performed at 23 weeks. It showed the following: multiple congenital anomalies (MACs) including fetal skin edema (HP:0025672), cerebral hypoplasia (HP:0006872), hand clenching (HP:0001188), contracture deformities in both upper joints (HP:0100360) and lower joint (HP:0005750) limbs, bilateral talipes (HP:0001776), decreased fetal movement (HP:0001558), hypertrophic cardiomyopathy (HP:0001639) with systolic dysfunction (HP:0006673), atrioventricular septal defect (HP:0006695).

Fetal U/S of the affected female sib was performed at 26 weeks and showed the following: MACs including skin edema, reduced fetal movements, cerebral hypoplasia, agenesis of corpus callosum (HP:0001274), hand clenching, contracture deformities in both upper and lower limbs, bilateral talipes, mild hepatomegaly (HP:0002240), hypertrophic cardiomyopathy, cardiomegaly (HP:0001640), and large atrioventricular septal defect.

**Fig. 1:**
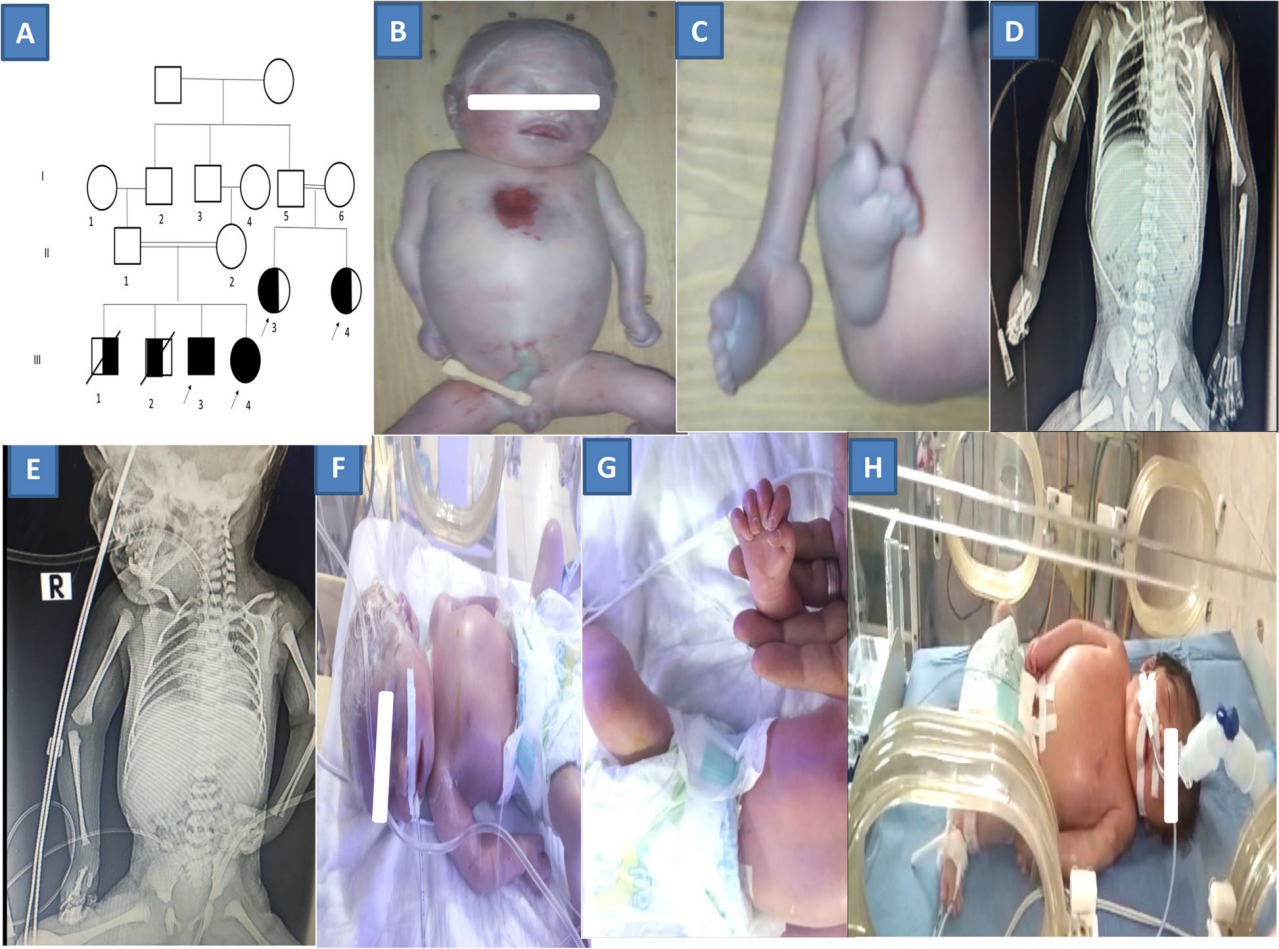
shows the Family Pedigree (**a**), external observed clinical manifestations (**b**, **c**) of the 3^rd^ (III-3) examined male sib; (**d**, **e**, **f**, **g**) show the clinical features and X-ray findings of the 4^th^ (III-4) female sib, and (H) show deceased (III-1) neonate without OCA features. Both (II-3 and II-4) were live female neonates with OCA features only

The third neonate (III-3) was a male patient with an age of 5 days when he was examined, and the fourth neonate (III-4) was a female patient of 7 days of age when she was examined. Both of them were presented phenotypically with features suggestive of oculocutaneous albinism. Both had the following: fair hair (HP:0002286), white eyebrow (HP:0002226), hypopigmentation of the skin (HP:0001010), ocular albinism (HP:0001107), hypertelorism (HP:0000316), bulbous nose (HP:0000414), and low-set ears (HP:0000369). Additional features were detected in these patients including; pectus excavatum (HP:0000767), clenched fist (HP:0001188), bilateral talipes equinovarus (TEV) deformity (HP:0001762), left pesplanovalgus deformity (HP:0008081), bilateral knee flexion deformity (HP:0006380), prolonged prothrombin time (HP:0008151), increased blood urea nitrogen (51–55 mg/dl) (HP:0003138), elevated circulating creatinine concentration (2.8–2.9 mg/dl) (HP:0003259), and non-obstructive hypertrophic cardiomyopathy (HP:0001639). Aplasia cutis congenita (HP:0001057) and absent sternum (HP:0006714) with visible cardiac contractions have been detected only in the 3^rd^ (III-3) neonate Fig. (1).

Anthropometric measurements for both neonates were normal for their age. The X-ray only revealed a right absent radius (HP:0011908) in the 4^th^ sib (III-4). Both of them died of congenital heart disease in the neonatal intensive care unit (NICU) at the age of 5 and 10 days respectively. There was a positive family history of two female patients (II-3 and II-4) who have OCA without cardiac affection. This family's apparent mode of inheritance was consistent with an autosomal recessive disorder.

### Karyotyping and CNV analyses:

**Fig. 2:**
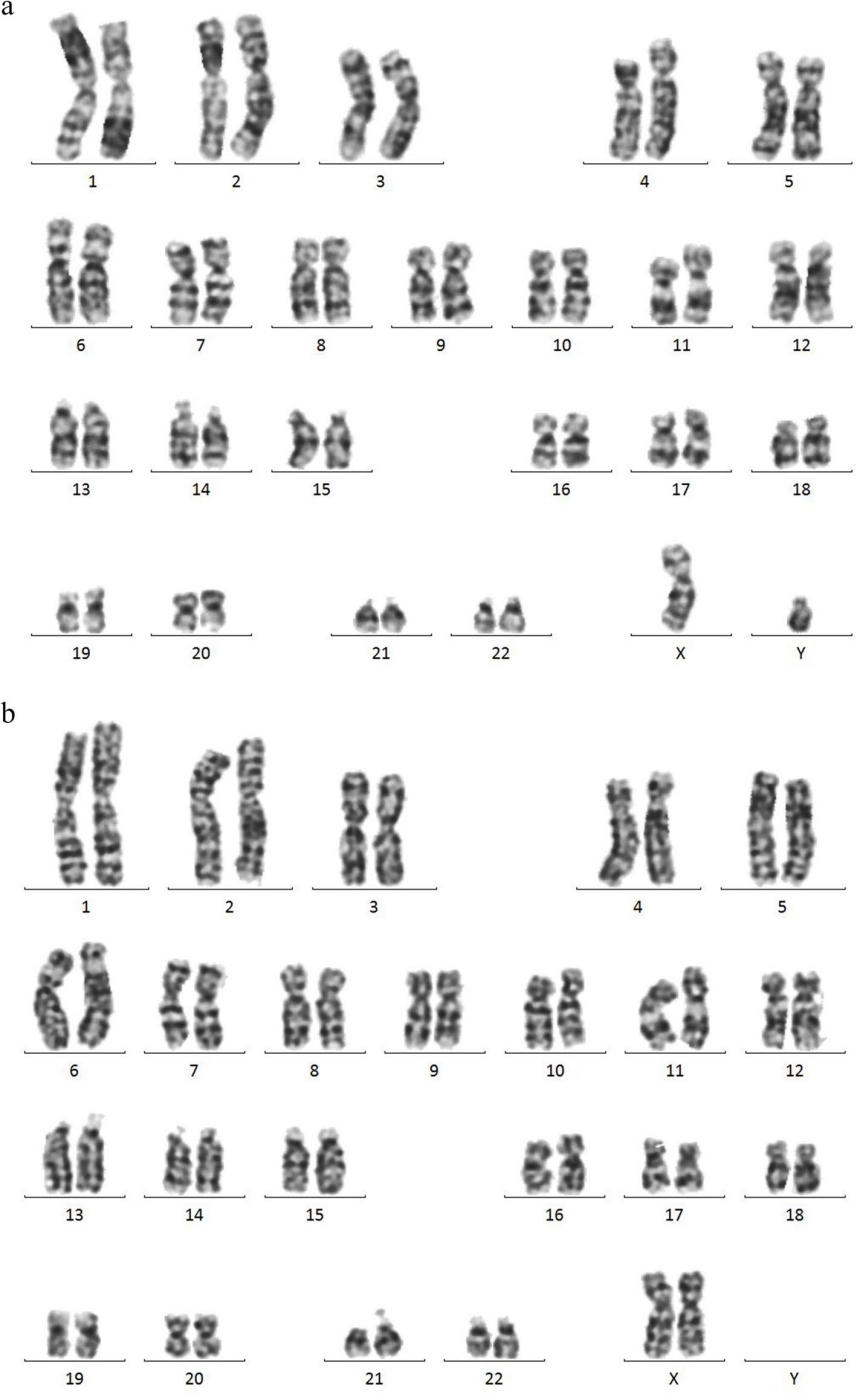
**A** Normal male karyotype of 3^rd^ neonate; 46, XY. **B** Normal female karyotype of 4^th^ neonate; 46, XX

Conventional cytogenetic analysis (CCA) for the 2 sibs (III-3 and III-4 neonates) revealed normal karyotypes in all the studied cells; 46, XY, and 46, XX for the male (III-3) and female (III-4) patients respectively Fig. 2 (A, B). Copy number variation (CNV) analysis was performed by whole exome sequencing (WES) covering chromosomal deletion/duplication abnormalities. Normal patterns were detected.

### Molecular results

Due to obvious clinical manifestations of OCA1, positive family history of OCA1 and to clarity of oculocutaneous albinism traits, we used Sanger sequencing to explore the nucleotide order of *the TYR* gene first. Subsequently, the following clinical examinations for III-3 and III-4 neonates led to the observation of additional new emerging symptoms that were not explained by known *TYR* etiology. Therefore, we did whole exome sequencing to explore other potentially relevant known disease-causing genes.

Using Sanger sequencing for whole coding exons of *the TYR* gene and its exon–intron boundaries in 3^rd^ (III-3) and 4^th^ (III-4) neonates, we detected NM_000372.5:c.346C > T p.(Arg116*) and NM_000372.5:c.446A > C p.(Tyr149Ser) variants in *TYR* gene. The subsequent WES for the same affected neonates showed NM_000372.5:c.346C > T p.(Arg116*) variant -(had previously been classified as a pathogenic variant in the *TYR* gene)- and the filtering pipeline prioritized a homozygous NM_015533.4:c.598G > A p.(Val200Ile) variant in *TKFC* gene. Both of these variants were in the expected homozygous state.

After the alignment of the patient's DNA reads to the UCSC human genome GRCh37/hg19, there were an average of 23,195 variations. After filtering out known SNPs using the Genome Aggregation Database, 1000 Genomes, and dbSNP databases, there was consensus of 9,325 variants. These variations were reduced to 3,101 after filtering for variants using the gnomAD database, and variants reported in HGMD and in ClinVar databases that have MAF > 1% and are likely to affect the protein sequence. With an emphasis on AR inheritance variants, the number of homozygous variants was reduced to 13. Further variants sorting according to the observed patients' features led to the identification of potentially relevant variants that were known variants in TYR and TKFC genes in each patient. No further known clinically relevant variants related to the provided clinical features including especially cardiomyopathy, metabolic disorders, and skeletal disorders were detected. The copy number variation (CNV) detection was normal, with a sensitivity of more than 95%.

### Segregation analysis results

**Fig. 3:**
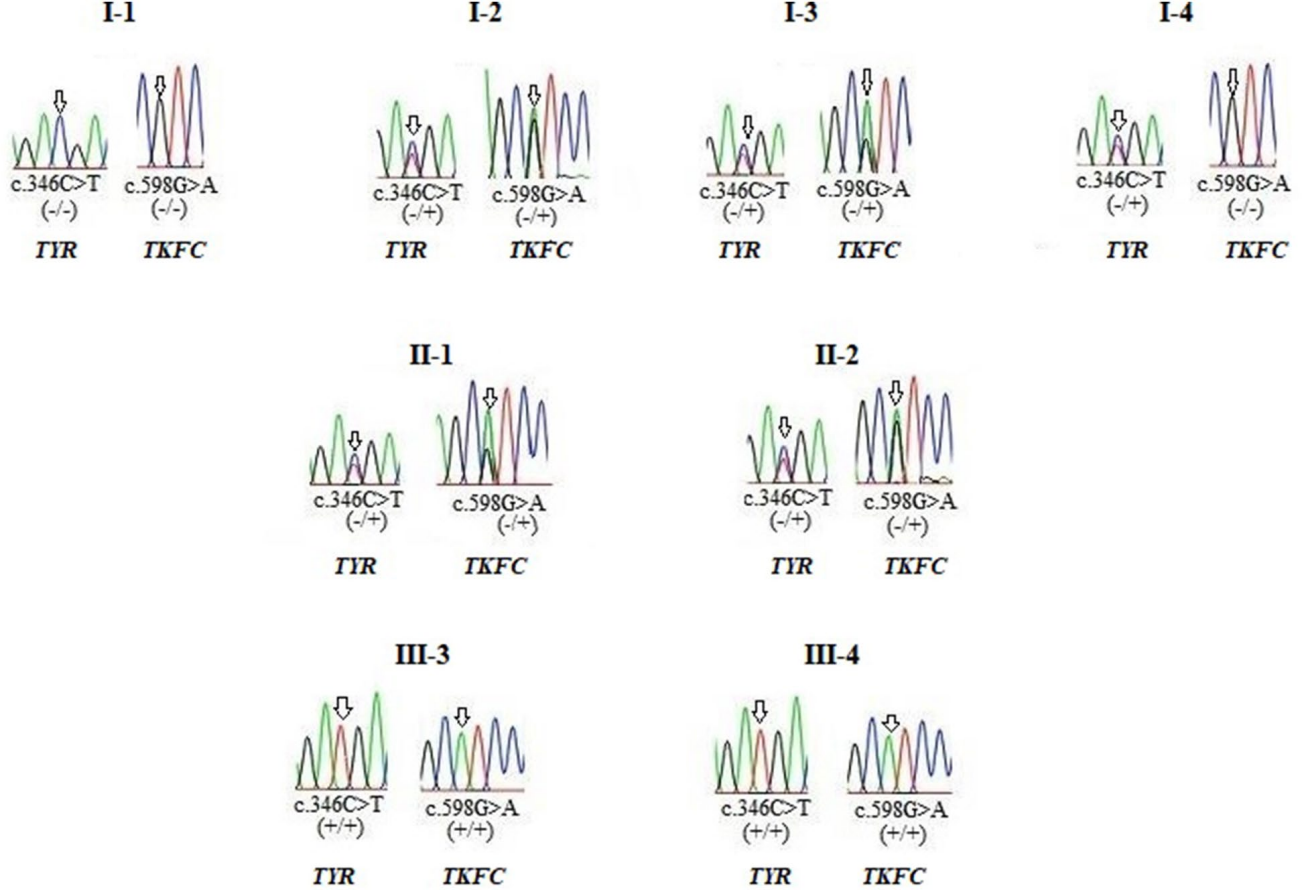
Positive co-segregating for c.346C > T(*TYR)* and c.598G > A(*TKFC)* variants. Beneath each sequence chromatogram, wild and mutant alleles are indicated by a—or + sign, respectively

Biallelic variants in *TYR* and *TKFC* were confirmed by Sanger sequencing and positive co-segregation data. The inheritance of c.346C > T (*TYR*) and c.598G > A (*TKFC*) explained complete penetrance across 8 family members in which co-existence of the two variants in 3^rd^ (III-3) and 4^th^ (III-4) neonates with homozygous state exist. Their parents (II-1 and II-2) were in heterozygous state, and variable inheritance states in their grandfathers (I-1 and I-2) and their grandmothers (I-3 and I-4). Therefore, the Pedigree of 3 generation family members of OCA1 showed co-segregated appropriately on Sanger sequencing chromatograms as shown in Fig. (3).

The segregation analysis showed that: (i) c.446A > C variant in *TYR* gene seems benign because it was detected in the unaffected family members e,g, grandfather in heterozygote state, in the grandmother in homozygote state, and absent in the affected members, (ii) variable inherited penetrance profile for c.346C > T (*TYR*) and c.598G > A (*TKFC*). Combined familial segregation analysis and the clinical features confirmed a clinical pathogenic impact of c.346C > T (*TYR*). It also indicates a potential clinical pathogenic impact of c.598G > A (*TKFC*) with an extended phenotypic spectrum. Therefore, the pattern of phenotypes within a family is consistent with c.346C > T (*TYR*) and c.598G > A (*TKFC*) transmission pattern. This finding confirms that the mode of inheritance is co-existence autosomal recessive (AR). To some extent, c.446A > C (*TYR*) is just an associated variant carrying an opposite *TYR* allele to *TYR*^c.346C>T^ allele. We have registered our observation concerning the following: (i) two probands' brothers (III-1 and III-2) who died shortly after the birth of hypertrophy cardiomyopathy (HCM) without marked OCA features, their peripheral blood can't be taken and their genotypes can't be detected, and (ii) positive family history of two female patients (II-3 and II-4) who have OCA without cardiac affection, their peripheral blood can't be taken and their genotypes can't be detected also. However, based on our segregation analysis results and consistency mode of inheritance in the family with autosomal recessive pattern, we reported the expected genotypes of those II-3, II-4, III-1, and III-2 patients for c.346C > T (*TYR*) and c.598G > A (*TKFC*) variants as shown in Fig. (3). The expected genotypes just can give supportive way for c.598G > A (*TKFC*) pathogenicity and role it in extension of *TKFC* variants spectrum, but it was not of definite evidence.

### Computational results

All the detected variants had been previously reported and classified as follows: c.346C > T (*TYR,* rs61753256) was classified as a pathogenic variant without a clear mode of pathogenicity, and c.598G > A (*TKFC*, rs149571662) was classified as uncertain significant (VUS). Except, c.446A > C (*TYR*, rs797046082) was not classified yet.

#### High NMD + sensitivity of TYR transcript harboring c.346C > T variant is a novel suggestive pathogenicity mechanism

c.346C > T is located in the first coding exon of *the TYR* gene, the biggest *TYR* exon, according to a canonical MANE transcript NM_000372.5. Clinical evidence for this variant was reported, where reputable sources recently reported the c.346C > T variant as pathogenic with extremely low frequencies in gnomAD = 0.000028 and TGP = 0.000024. To predict the pathogenicity mode of this variant, we used multiple lines of computational tools. Nonsense-mediated decay (NMD) analysis using the MutationTaster (MT) tool pointed to that this variant might lead to truncated protein with high NMD + transcript susceptibility. Therefore, c.346C > T could be classified as a loss of function (LoF) variant supporting its positive clinical pathogenicity evidence.

According to the ACMG classification guideline, TYR; c.346C > T variant can be classified as pathogenic as it matches criteria PVS1 (very strong evidence of pathogenicity; null variants are known mechanism of disease), PP5 (pathogenic supporting; a reputable source recently reports variant as pathogenic, but the experimental functionality evidence is absent), PM2 (pathogenic moderate; Extremely low frequencies in the gnomAD population and TGP databases), and PP1 (pathogenic supporting; positive co-segregation).

#### Alternative conformational mobility of L2-K1/TKFC is novel molecular dynamic-based supportive pathogenicity evidence for the c.598G > A variant

c.598G > A is located in the seventh coding exon of the *TKFC* gene according to a canonical MANE transcript NM_015533.4. No clinical evidence was previously explored for this variant. However, it has extremely low frequencies in gnomAD = 0.00014 and TGP = 0.00020. Annotation and conservation analysis showed that this variant is located within the DhaK (K) domain with a highly conserved score (PhastCons = 0.999 & PhyloP = 4.027). The alignment of the amino acid showed that val^200^ resided in a highly evolutionary conserved region. Pathogenicity prediction of c.598G > A showed benign matrices according to SIFT, PolyPhen-2, CADD, REVEL, MetaLR, and MutationAssessor. Whereas, MutationTaster (MT) showed its disease-causing impact based on its probable marginally splicing effect increasing acceptor site scoring.

*TKFC* has not been registered in experimental crystallography images. Using Phyre^2^ and AlphaFold DB, the full canonical MANE-based *TKFC* protein was homology modeled on the crystal structure of the dihydroxyacetone kinase from the Citrobacter freundii template (PDB ID 1UN9). *TKFC* was modeled based on heuristics to maximize confidence, percentage identity, and alignment coverage. Reliability and quality of modeled *TKFC* were performed using the Qualitative Model Energy ANalysis Distance Constraints (QMEANDisCo) tool showing a global confidence score of 0.73 ± 0.05 and a confidence score of Val^200^ was 0.76. The modeled *TKFC* showed that almost the secondary structure of *TKFC* was not altered by c.598G > A p.(val200Ile) variant. Both valine (Val) and isoleucine (Ile) are non-polar hydrophobic amino acids located as coil structure (disordered structure) at the surface of *TKFC* enzyme. Presence of valine residue on *the TKFC* surface evokes us to compute differential physiochemical distance and fluctuation/conformational flexibility scores between valine and isoleucine. No significant physiochemical distance between the Val and Ile was found.

**Fig. 4:**
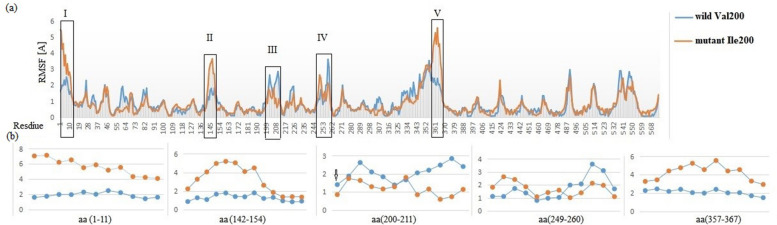
Differential fluctuation regions between wild^Val200^ and mutant^Ile200^ peptide chains of the *TKFC*. **a** Root-mean square fluctuations (RMSF) of Cα atom positions relative to their mean positions in wild (blue highlighted) and mutant (red highlighted) showing the main five highly fluctuation regions, where all the detected regions show high fluctuation due to a mutant form except region (III) that exhibits low fluctuation pattern of a mutant form. Region (IV) shows struggle fluctuation. **b** detailed fluctuating regions show differential fluctuating amino acid coordinates. Arrow refers to p.(Val200Ile) position

**Fig. 5:**
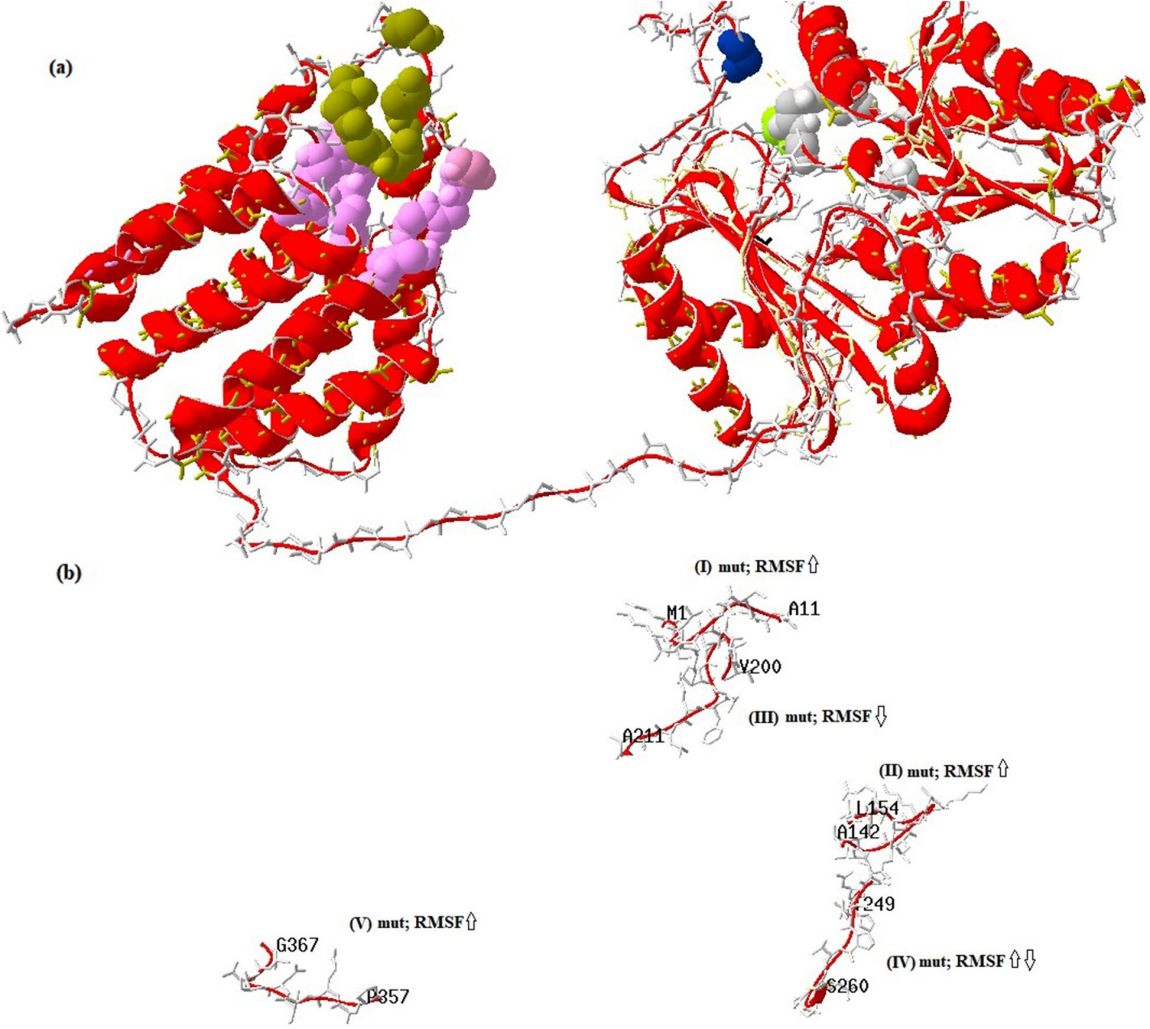
**a** Homology model of human L2-K1/*TKFC* in complex with dihydroxyacetone (DHA) on the K1 subunit, and adenosine triphosphate (ATP) on the L2 subunit. Open L2-K1 active site conformation showing a too large distance which potentially prevents a phosphoryl transferring from ATP molecules to DHA compounds. The position of p. (Val200Ile) is blue highlighted. Positional annotations of ATP, DHA, fluctuating amino acids, Val^200^, and His^221^ were derived from the UniProt (Q3LXA) database. **b** Corresponding fluctuating regions, in which disordered structured regions show dominant sensitivity to fluctuation alteration. The upper arrows represent a high-fluctuating structure due to a mutant form, and the lower arrows represent low fluctuating structure due to a mutant form. Together upper and lower arrows refer to struggle fluctuation structure

After energy minimization of wild^Val200^ and mutant^Ile200^ TKFC model using the YASARA tool, the physical contact of residues realism and geometry of side-chain accuracy were improved. Then, the prediction of differential fluctuating residues and regions between wild^Val200^ and mutant^Ile200^ TKFC models were computed using the CABSflex tool showing five highly altered fluctuating regions. It also showed that Isoleucine is less flexible than valine with scores of 0.858 and 1.408 respectively as shown in Fig. (4). By annotating the highly fluctuating amino acids on *TKFC* model, we found that Val^200^ resides in highly fluctuating Ser-Pro rich region on K1 domain, and on the opposite site of L2 domain highly fluctuating Ala-Gly rich region was found. The location of the regions and amino acids numbers with higher and lower fluctuation in the structural model of TKFC is shown in Fig. (5). From the fluctuation/conformational flexibility results, we assumed pathogenicity of c.598G > A; p.(Val200Ile) variant to altered root-mean-square fluctuation (RMSF) effect resulting from lower fluctuation of Ile than Val by 0.55 A^o^, and consequently this variant might lead to rigidity of L2-K1/TKFC active site.

c.598G > A is previously reported as VUS. According to ACMG classification guideline, c.598G > A variant can be classified as VUS as it matches criteria PM2 (pathogenic moderate; Extremely low frequencies in gnomAD population and TGP databases), PP1 (pathogenic supporting; positive co-segregation), and BP4 (benign supporting; computational prediction tools unanimously support a benign effect).

### Pathway enrichment analysis of TKFC

To explore first-tier reactants for *TKFC*, we used the STRING tool to construct a protein–protein interaction (PPI) network showing experimental evidence to interact Interferon-induced helicase C domain-containing protein 1 (*IFIH1*) with *TKFC* with interaction score = 0.906 under highest confidence = 0.900. IFIH1 is involved in the innate immune system releasing type 1 interferons and proinflammatory cytokines. Our KEGG pathway analysis showed that *TKFC* is enriched in the RIG-I-like receptor signaling pathway leading to elevated inflammatory cytokines that are; IL-8, TNF alpha, IL-12, and IP10.

Here, pathogenicity evidence of c.346C > T (*TYR*) and c.598G > A (*TKFC*) variants included positive co-segregation data committed with co-existence AR inheritance, very low MAFs, ACMG classification of c.346C > T (*TYR*) variant, molecular dynamic (fluctuation) results of c.598G > A (*TKFC*).

## Discussion

This study demonstrated two affected neonates with OCA1 and TKFC-relevant traits who died of congenital heart disease in the NICU at the age of 10 days. We could not perform many recommended assays like mitochondrial disorder markers and muscle histology. Positive co-segregation for overlapped clinical traits relevant to *TYR* and *TKFC* variants was demonstrated.

The human *TYR* gene is composed of five exons encoding tyrosinase, a copper-containing oxidase [6]. Tyrosinase is incorporated in the first two reactions of the melanin biosynthesis pathway, so a defect in *TYR* causes a complete or partial lack of melanin [15]. Most of the *TYR* variants were null variants located in two copper-binding sites, 39- end of the copper B-binding region near the N terminal region, and between the CuA and CuB domains [1].

*TKFC* encodes a bifunctional protein that has been annotated as a homodimeric triokinase (K domain) that catalyzes the adenosine triphosphate (ATP)-dependent phosphorylation of GA. DHA, and FMN cyclase (L domain) that catalyzes internal cycling reaction of Mn^2+^-dependent splitting of FAD to form AMP and cyclic FMN. K and L domains order in intertwined subunits forming elongated L2-K1-K2-L1 structure [7, 8].

In this study, we identified three reported variants within *TYR* and *TKFC* genes in OCA1 family members, and the affected probands presented with atypical clinical features. *The current two OCA1 neonates are the first worldwide registered patients under individual LOVD ID 00407086 (Phenotype #0000299473).* The data supporting the study are available at https://databases.lovd.nl/shared/individuals/00407086. We detected homozygous c.346C > T and c.446A > C variants in *the TYR* gene, and homozygous c.598G > A variant in *the TKFC* gene. Both c.346C > T and c.446A > C lie in the intramelanosomal domain (IMD; residues 19–476) of the *TYR* gene with a binuclear copper binding site. c.598G > A is located in the K domain in the N-terminal of *TKFC*.

Our segregation analysis demonstrated positive co-segregation for co-existence AR inheritance of homozygous c.346C > T and c.598G > A variants in TYR and *TKFC* respectively. Positively co-segregation data supported that the c.346C > T variant is alone responsible for the presented autosomal recessive OCA1 features. c.598G > A variant is potentially responsible for the presented extra autosomal recessive non-OCA1 features.

c.346C > T variant was only presented with a homozygosity state in the affected neonates and a heterozygosity state in some unaffected family members. Nonsense-mediated decay (NMD) prediction analysis showed that the transcript containing c.346C > T variant is sensitive to NMD^+^ based on a 50-bp rule for transcript degradation prediction with premature termination codons (PTCs) relative to the last exon-exon junction (EEJ) [16]. Liu et al. [17], Shah et al. [18], and Sun et al. [6] pointed out that the c.346C > T variant has a deleterious impact and causes OCA1, [6, 17, 18]. Lin et al. [1] pointed out that c.346C > T variant have been described as a pathogenic variant in Caucasian OCA1 patients [1]. Therefore c.346C > T variant might be a key pathogenic variant in our OCA1 patients.

Although c.598G > A in the *TKFC* gene was previously registered as a VUS variant, our multiple sequence alignment analysis showed that the valine residue is highly conserved in evolution. Hence, it has a deleterious effect is reasonable. This prediction is consistent with what was mentioned by Chan et al. [19] that conserved amino acids had a 91.6% to 96.8% chance of being deleterious [19]. The probability of positive pathogenicity of this variant was also supported by positive co-segregation and molecular fluctuation analyses.

More recently, it was found that *TKFC* variants, Gly445Ser and Arg543Ile, are associated with cataracts and multisystem disease [8]. Also, Gly192Arg and Arg228Trp are associated with autosomal recessive hypotrichosis with loose anagen hairs [20]. Wortmann et al. [8] performed trio genome and exome sequencing on two families presenting multisystem disease traits which were fatal dilated cardiomyopathy with poor systolic function, abnormal liver function with hypoalbuminemia, and metabolic inborn error with lactic acidosis in a patient harboring the c.1628G > T variant (located within the FMN lyase domain) in* TKFC* gene [8]. Hypertrophic Cardiomyopathy with systolic dysfunction, liver dysfunction, and metabolic inborn errors was observed in our affected neonates. According to our clinical findings, the deceased OCA1 neonates had prolonged prothrombin time indicating liver dysfunction, and increased blood urea and creatinine concentrations indicating metabolic errors. It is worth noting that HCM related to *TKFC* dysfunction was previously observed according to the Orphanet database (ORPHA:1369).

In this study, molecular dynamics analysis showed that wild Val^200^ is a more fluctuating amino acid than mutant Ile^200^. This differential fluctuation between Val and Ile may lead to a partial lack of transfer of a phosphoryl group of ATP molecule on the L domain to trios on the K domain. Hence, decreased phosphorylated GA and DHA may lead to the accumulation of a highly reactive aldehyde group. Rodrigues et al. [7] showed an open conformation structure between the L2-K1 structure containing an active site to transfer a phosphoryl group from ATP (on the L domain) to DHA (on the K domain). The same authors found that this open conformation structure is the too large distance (≈14 Å) for transferring the phosphoryl group. They pointed out that Val^200^ locates in a highly fluctuated peptide which is necessary to decrease the distance between ATP and DHA permitting ATP-dependent phosphorylation of DHA to occur. They stated that this fluctuated peptide has a major role to decrease of the open conformation between L2 and K1 subunits from 14^o^ to 5A^o^ [7].

Interestingly, our fluctuation analysis showed that five highly differential fluctuating regions resulted from mutant^Ile200^ TKFC, one of them including residues from 200 to 211 codons where p.(Val200Ile) resided leading to a lower fluctuation region than the wild form. According to what was mentioned by Rodrigues et al. (2019), p.(Val200Ile) is a highly potential causative variant to decrease L2-K1 structure closure, and hence it is proposed to be an obstacle for ATP-dependent phosphorylation of DHA decreasing and excessive aldehyde accumulation [7].

From the given above, we can conclude that the pathogenicity of the c.598G > A p.(Val200Ile) variant is due to the presence of less fluctuated Isoleucine amino acid. Thus, Ile^200^ may lead to rigid open conformation between L2 and K1 subunits preventing phosphorylation of GA and DHA. Subsequently, the lack of GA and DHA phosphorylation is a potential risk for the accumulation of aliphatic aldehyde compounds containing highly reactive carbonyl groups.

Atik et al. [21] and Wortmann et al. [8] pointed out that *TKFC* deficiency leads to impaired fructose catabolism leading to excessive glyceraldehyde formation. Glyceraldehyde is a reactive molecule that may interact with multiple proteins producing reactive oxygen species (ROS) and subsequent inflammation responses [8, 21]. Also, Xu et al. [22] revealed that increased serum levels of aldehydes are associated with kidney disease, liver disease, lipid metabolism, inflammation, and heart failure disease [22]. Results of the Xu et al. [22] study support some additional non-OCA1 traits that was observed in this study. We observed in our enrolled affected neonates the following: kidney failure markers with elevated circulation urea nitrogen and creatinine, liver dysfunction with prolonged prothrombin time and elevated C-reactive protein (CRP), and heart disease.

O'Brian et al. [23] pointed out that CRP is an inflammatory biomarker and is controlled by pro-inflammatory cytokines, mainly IL-6, IL-1, and TNF [23]. Yang et al. [24] found that elevated CRP in serum is closely associated with urea and creatinine elevation because of its role in increasing the molecule adhesion in the kidney, besides it represents a high risk of heart attacks [24]. Therefore, we can hypothesize that elevated CRP in our affected neonates was before urea and creatinine elevation. Also, elevation of CRP, urea, and creatinine might be risk factors for hypertrophic cardiomyopathy occurrence.

Our PPI analysis showed a strong interaction score between *TKFC* and IFIHI supported by experimental evidence. Recently, *TKFC* has been a negative regulator of IFIH1 which is involved in RNA and virus-mediated type I interferon (IFN) production and antiviral responses [26–28]. Crow et al. [28], Rutsch et al. [29], and Amari et al. [30] presented variability phenotypes relevant to IFIH1 dysfunction including skeletal abnormalities (osteoporosis, distal limb osteolysis, widened medullary cavities, and lower limb spasticity), facial dysmorphism (high anterior hair line, broad forehead, smooth philtrum, thin upper vermilion border), Cerebral atrophy, and cardiomegaly [29–31]. Interestingly lower limb spasticity, cerebral deformity, and cardiomegaly were observed in our index patients.

Taking all of these findings into consideration, we can postulate that the observed non-OCA1 traits may be due to c.598G > A through two mechanisms: (i) accumulation of highly reactive glyceraldehyde. This mechanism caused elevation of urea, creatinine, prothrombin time, CRP, and heart disease. The 2nd mechanism could be (ii) through the gain of function IFIH1. This mechanism led to cerebral hypoplasia and skeletal deformities. However, the complete *TKFC* function is still unclear to a certain extent, where variable clinical traits are still emerging. Although our results indicated expanded features for a detected *TKFC* variant (Table 1), extended experimental analysis is recommended. The observed positive parental consanguinity could increase the incidence of recessive genetic disorders, [31].

**Table 1:** Cardinal phenotypic features due to *TKFC* mutation compared to expanded features in our study

Phenotypic features due to *TKFC* variant^c^	Reported *TKFC* features^a^	*TKFC* Features in Our Patients	*TKFC* Features in our patients as expanded clinical findings of *TKFC* variant
Multisystem disorders	+	+	
Failure to thrive	+	-	-
Congenital cataract	+	-	-
Microphthalmia	+	-	-
Hypertrophic cardiomyopathy (HP:0001639)	+	+	-
Poor systolic function (HP:0006673)	+	+	
Abnormal liver function	+	( +) Prolonged prothrombin time (HP:0008151)	-
Pancreatic affection	+	-	-
Developmental delay	+	( +) Decreased fetal movement (HP:0001558)	-
Fatty degeneration of the liver	+	NA^b^	-
Hepatomegaly (HP:0002240)	+	( +) mild	-
Cerebellar hypoplasia (HP:0006872)	+	+	-
Lactic acidosis	+	-	-
muscle weakness	+	NA	-
**New phenotypic features**			
Pectus excavatum (HP:0000767)	**-**	+	+
Hand clenching (HP:0001188)	**-**	+	+
Aplasia/Hypoplasia of the sternum (HP:0006714)	**-**	+	+
Talipes equinovarus (HP:0001762)	**-**	+	+
Unilateral radial aplasia (HP:0011908)	**-**	+	+
Knee flexion contracture (HP:0006380)	**-**	+	+
Aplasia cutis congenita (HP:0001057)	**-**	+	+

^a^According to the registered features in OMIM and Orphanet databases

^b^NA: Not applicable due to very young age and early sudden death of the cases

^c^HPO IDs are written for the observed features only

## Conclusions

We identified the co-existence of *TYR* and *TKFC* variants in atypical OCA1 neonates with severe skeletal abnormalities and fatal hypertrophic cardiomyopathy. Sequencing and segregation analysis revealed that both c.346C > T in *TYR* and c.598G > A in *TKFC* could cause non-syndromic OCA1 combined with skeletal abnormalities and fatal hypertrophic cardiomyopathy. Supportive molecular dynamics results provided potential molecular pathogenesis evidence of c.598G > A p.(Val200Ile) in *TKFC,* where Ile residue is partially preventing closure of the open conformation interface between L2 and K1 subunits. The utility of *TKFC* sequencing to differentiate TYR-positive OCA1 patients with fatal cardiomyopathy and skeletal deformities is recommended. Additionally, we can conclude that analysis of circulating urea/creatinine/aldehydes levels, and echocardiography for findings of cardiomyopathy may be considered as fast differential diagnostic markers.

## Data Availability

All data are available at Clivar Database: https://www.ncbi.nlm.nih.gov/clinvar/variation/99565/ Classification and reserved accession number SCV005091107 for submission SUB14637329. Classification and reserved accession number SCV005091108 for submission SUB14637353. Classification and reserved accession number SCV005091106 for submission SUB14637297. Under the following Links: https://www.ncbi.nlm.nih.gov/clinvar/?term=TKFC+c.598G%3EA+p. https://www.ncbi.nlm.nih.gov/clinvar/?term=TYR+c.446A%3EC+p. https://www.ncbi.nlm.nih.gov/clinvar/?term=TYR+c.346C%3ET+p. However, other more data supporting the results of this article are available upon request.
